# A network-based method for associating genes with autism spectrum disorder

**DOI:** 10.3389/fbinf.2024.1295600

**Published:** 2024-03-08

**Authors:** Neta Zadok, Gil Ast, Roded Sharan

**Affiliations:** ^1^ Blavatnik School of Computer Science, Tel Aviv University, Tel Aviv, Israel; ^2^ Department of Human Molecular Genetics and Biochemistry, Sackler Faculty of Medicine, Tel Aviv University, Tel Aviv, Israel

**Keywords:** autism spectrum disorder (ASD), network propagation, machine learning, ASD genes, random forest

## Abstract

Autism spectrum disorder (ASD) is a highly heritable complex disease that affects 1% of the population, yet its underlying molecular mechanisms are largely unknown. Here we study the problem of predicting causal genes for ASD by combining genome-scale data with a network propagation approach. We construct a predictor that integrates multiple omic data sets that assess genomic, transcriptomic, proteomic, and phosphoproteomic associations with ASD. In cross validation our predictor yields mean area under the ROC curve of 0.87 and area under the precision-recall curve of 0.89. We further show that it outperforms previous gene-level predictors of autism association. Finally, we show that we can use the model to predict genes associated with Schizophrenia which is known to share genetic components with ASD.

## Introduction

Autism spectrum disorder (ASD) is a complex neurological and developmental disorder that affects a person’s behavior, communication, and learning abilities. About 1 in 44 children is identified with the disorder according to estimates from Centers for Disease Control and Prevention (CDC) Autism and Developmental Disabilities Monitoring (ADDM) Network (Maenner, 2021). It is thought to be caused by a combination of genetic and environmental factors that impacts the structure and function of the brain and nervous system (Flickr, 2023). Identifying the genetic base of ASD is critical to understanding its underlying biological mechanisms. Such knowledge will impact the development of new interventions and treatments for individuals affected by this disorder.

Extensive molecular studies have charted the landscape of ASD with respect to different information layers including genome wide association (GWAS), differential gene expression (Parikshak et al., 2016; Gandal et al., 2018), differential transcript expression (Gandal et al., 2018), alternative splicing changes (Parikshak et al., 2016; Gandal et al., 2018), differential methylation (Wong et al., 2019), copy number variation (Sanders et al., 2015) and more (Satterstrom et al., 2020). Each of these studies have come up with candidate lists of ASD-associated genes, calling for computational methods to consolidate these gene lists.

Machine learning based methods offer a new perspective to the problem by learning from known ASD-related genes and building models that provide ways to prioritize the risk associated with previously unknown genes based on their predicted scores (Liu et al., 2014; Krishnan et al., 2016; Duda et al., 2018; Brueggeman et al., 2020; Lin et al., 2020). These methods differ in their training features and prioritization method. (Duda et al., 2018) and (Krishnan et al., 2016) leveraged brain-specific functional interaction networks to produce genome-wide rankings of ASD associated genes. (Liu et al., 2014) clustered evidence for ASD association within a co-expression network in specific brain regions. (Lin et al., 2020) used features extracted from spatiotemporal gene expression patterns in the human brain. Last, the state-of–the-art forecASD (Brueggeman et al., 2020) integrates network-based information from large gene interaction networks with scores of genetic association and brain gene expression information. In detail, novel features are generated from BrainSpan expression and STRING interaction data. These are combined with literature-derived features from DAWN, DAMAGES, and Krishnan (Liu et al., 2014); (Krishnan et al., 2016; Zhang and Shen, 2017) and used to train a random forest classifier for ASD association.

The methods described above have predominantly relied on a single data source, potentially missing relevant information. Conversely, some approaches such as forecASD utilize multiple data sources that are devoid of network context. To address these limitations, our proposed method leverages a network propagation technique to integrate diverse data sources while accounting for their network context. Our model employs network-propagation on ASD associated genes from different data sets to derive predictive gene scores. Features are then combined using a random forest classifier. We evaluate the performance of our model and compare it to previous methods. Finally, we use our model to predict Schizophrenia-associated genes.

## Materials and methods

### Classification pipeline

**FIGURE 1:**
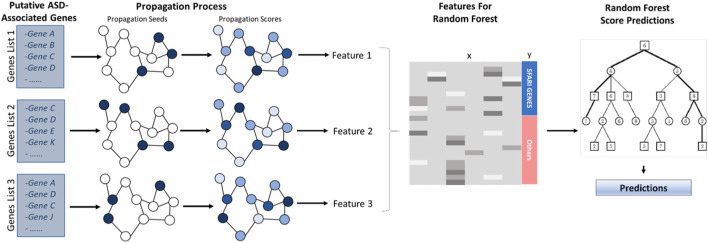
ASD Classifier Flowchat. *Putative ASD-associated genes serve as seeds to a network propagation process. After propagation, the resulting scores yield classification features for a random forest classifier that is used to compute association scores.*

The computational pipeline has two stages. First, network-based gene features are generated using a network propagation technique. Second, a random forest model is applied to these features to yield the prediction score. The classifier is summarized in Figure 1. Its stages are described in the following sections.

### Feature generation

Our starting point is gene lists obtained from the literature as detailed in Table 1. Overall, we use ten gene sets that were suggested to be associated with ASD based on various layers of information.

**TABLE 1:** Lists of ASD associated genes according to different sources.

	References	Data type	Data source	Number of genes
1	Gandal et al., (2018)	Differential gene expression (DGE)	Frontal and temporal cortex brain samples	1,611
2	Gandal et al., (2018)	Differential transcript expression (DTE)	Frontal and temporal cortex brain samples	767
3	Gandal et al., (2018)	Summary-data–based Mendelian randomization (SMR)	Frontal and temporal cortex brain samples	36
4	Gandal et al., (2018)	Transcriptome-wide association study (TWAS)	Frontal and temporal cortex brain samples	12
5	Parikshak et al., (2016)	Differential gene expression (DGE)	Cortex Samples of frontal and temporal cortex and cerebellum	1,142
6	Parikshak et al., (2016)	Differential alternative splicing analysis	Cortex Samples of frontal and temporal cortex and cerebellum	833
7	Satterstrom et al., (2020)	Transmitted and *de novo* association (TADA) model	Whole-exome sequence (WES) data from 35,584 samples	102
8	Wong et al., (2019)	Cross-cortex iASD-associated genes that reported both signficant differential DNA mtheyltaion and transcriptional changes	223 post-mortem tissues samples isolated from three brain regions [prefrontal cortex, temporal cortex and cerebellum (CB)]	18
9	Wong et al., (2019)	Cross-cortex dup15q-associated genes that reported both significant differential DNA methylation and transcriptional changes	223 post-mortem tissues samples isolated from three brain regions [prefrontal cortex, temporal cortex and cerebellum (CB)]	74
10	Sanders et al., (2015)	Analysis of *de novo* CNVs (dnCNVs) from the full Simons Simplex Collection		65

Each of these ASD related gene lists is used as a seed for a network propagation process that pinpoints other genes with high proximity to the seed set in a protein-protein interaction (PPI) network. The initial value of each seed protein from a list of size *s* is set to *1/s*. We use a human PPI network from (Signorini et al., 2021) which has 20,933 proteins and 251,078 interactions in its main connected component. We run network propagation with default damping parameter ɑ = 0.8. We normalize the results using the eigenvector centrality method (Erten et al., 2011) in order to avoid biases which are caused by the degrees of the proteins. The resulting ten propagation scores for each gene comprise its feature set.

### Random forest model

The features of a gene are integrated using a random forest model. To train the model, we use SFARI’s Gene Scoring Module (Abrahams et al., 2013) which offers critical evaluation of the strength of the evidence for each gene’s association with ASD. The genes are assigned to four categories: “Syndromic” (S), “Category 1” (High Confidence), “Category 2” (Strong Candidate) and “Category 3” (Suggestive Evidence). We label “Category 1” genes as positives (206 in total) and randomly pick 206 negative genes that do not appear in the SFARI database.

The random forest model is trained with the “sklearn” Python package using its default parameters which are a maximum of 100 trees, no maximum tree depth and minimum number of samples required to split an internal node of 2.

## Results and discussion

### Model performance

**FIGURE 2:**
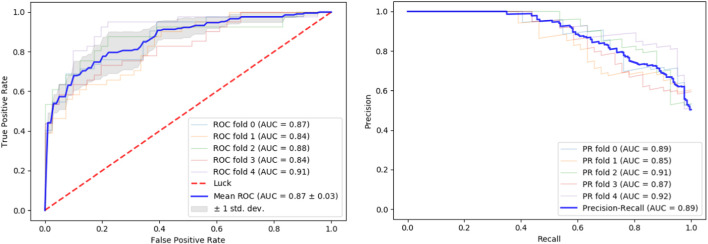
Performance evaluation. Performance of our classifier in 5-fold cross validation. The result of each fold and its mean are shown. Left: ROC curves. Right: Precision-recall curves.

We tested our classifier using 5-fold cross-validation. Figure 2 depicts ROC and precision-recall graphs showing the final result as the mean between all the five fold scores. The AUROC is 0.87 and the AUPRC is 0.89 indicating the high accuracy of our method.

To facilitate the model’s application by potential users, we calculated an optimal classification cutoff of 0.86, which maximizes the product of specificity and sensitivity (Liu, 2012). The full model scores can be found in Supplementary Table S2.

**FIGURE 3:**
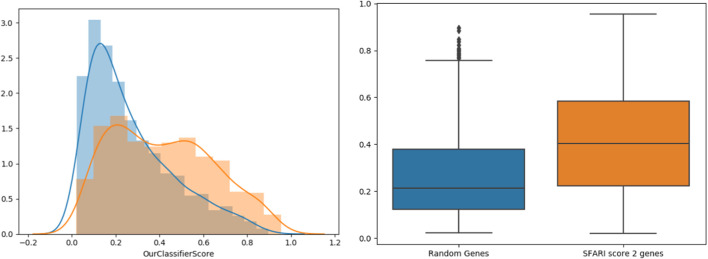
Classifier validation. Histograms (left) and box-plots (right) for SFARI (scores 2 and 3, orange) vs. random genes (blue).

To further support our results, we tested our classifier’s predictions on SFARI genes of scores 2 and 3 (which were not used during training), compared to randomly chosen negative genes (Figure 3). The classifier’s score distributions of the groups were compared using the Wilcoxon signed-rank test. Reassuringly, SFARI genes with scores 2 and 3 got significantly higher scores than the random negative genes (*p*-value < 3.62e-34).

**FIGURE 4:**
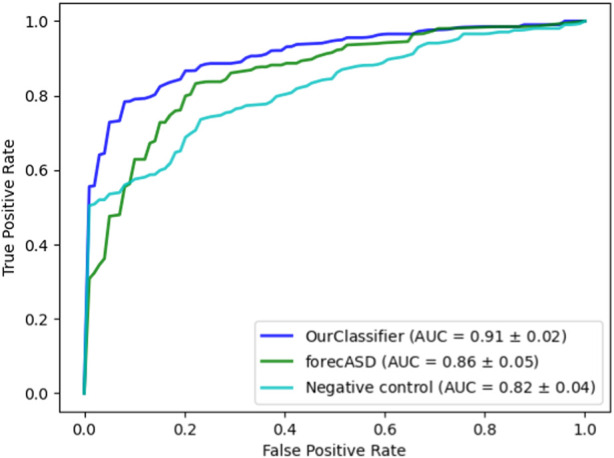
Performance comparison against forecASD and a negative control. Shown here are the mean ROCAUC results of each classifier.

After establishing the accuracy of our proposed method, we conducted a comparative analysis with the state-of-the-art forecASD classifier, which was shown to outperform earlier predictors. For this purpose, we employed the same random forest classifier on both our dataset and the features suggested by forecASD, namely BrainSpan and STRING. We additionally assessed the propagation procedure on a random degree-preserving network to act as a negative control. The results are provided in Figure 4 showing the superiority of our method compared to forecASD and the negative control (AUROC of 0.91 vs. 0.87 and 0.82). Notably, the relatively high AUROC of the negative control testifies to the quality of the gene sets that serve as seeds for the network propagation.

### Functional annotation analysis

**FIGURE 5:**
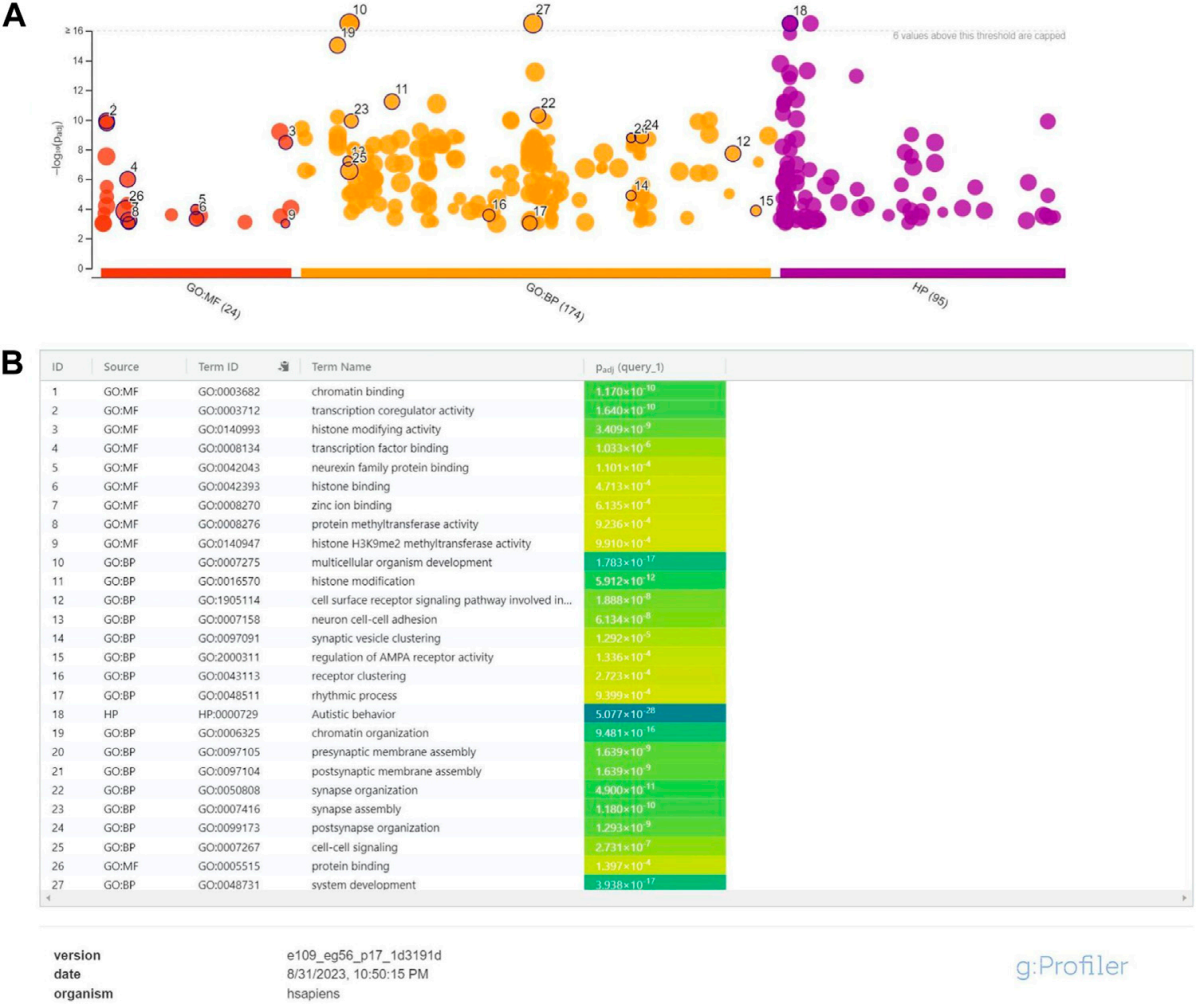
Functional annotation results for genes predicted by our classifier. **(A)** Manhattan plot created with g:Profiler illustrates the enrichment analysis results. The x-axis represents functional terms that are grouped and color-coded by data sources. The y-axis shows the adjusted enrichment *p*-values in negative log10 scale. Highlighted points in the plot are the terms which got the highest scores, and highlighted driver terms in GO created by g:Profiler algorithm. The algorithm is used for filtering GO enrichment results, providing a more efficient and reliable approach compared to traditional clustering methods. **(B)** Detailed information about highlighted circles from the Manhattan graph. Detailed information include data source, id and name of the term together with corresponding *p*-value.

Next, we wished to analyze the functional roles of the top predicted genes. To this end, we set a prediction threshold of 0.947, which maximizes the sum of precision and recall, and focused on the 84 genes passing this threshold. We conducted functional enrichment analysis using g:Profiler (version e109_eg56_p17_1d3191d) with Bonferroni corrected *p*-values and a significance threshold of 0.001 (Raudvere et al., 2019). The analysis is based on several data sources (GO:MF, GO:BP, Human Phenotype Ontology) (Figure 5).

From Human Phenotype Ontology—Autistic Behavior was the highest enriched phenotype. Using GO:BP (Biological Process) and GO:MF(Molecular Function) data sources, yield several highly enriched pathways known to play important roles in autism etiology including chromatin organization and binding (Haddad Derafshi et al., 2022), histone modification (Sun et al., 2016), neuron cell-cell adhesion (Eve et al., 2022) and zinc ion binding (Walsh et al., 2001; Walsh, 2002; Wang and Zhou, 2010; Yasuda et al., 2011). The full list of the functional annotation analysis results can be found in Supplementary Table S1.

### Exploiting schizophrenia genes

Given the known phenotypic similarity between ASD and schizophrenia (SCZ) (Hommer and Swedo, 2015) we wished to test whether our classification model could be further improved by adding information on SCZ associated genes. Thus, we collected lists of genes that were associated with SCZ (Table 2) and reapplied our computational pipeline.

**TABLE 2:** Lists of SCZ associated genes.

	References	Method	Data source	Number of genes
1	Gandal et al., (2018)	Differential gene expression (DGE)	Frontal and temporal cortex brain samples	4,821
2	Gandal et al., (2018)	Differential transcript expression (DTE)	Frontal and temporal cortex brain samples	3,803
3	Gandal et al., (2018)	Transcriptome-wide association study (TWAS)	Frontal and temporal cortex brain samples	193
4	Huang et al., (2020)	equality of variances to normalized expression data (evQTLs) Differential gene expression	dorsolateral prefrontal cortex of individuals affected with SCZ 212 SCZ and 214 unaffected control (CTL) samples	88
5	Huang et al., (2020)	expression variability QTL (evQTL) mapping analysis	dorsolateral prefrontal cortex of individuals affected with SCZ 212 SCZ and 214 unaffected control (CTL) samples	1,453

Indeed, the model based on both ASD, and SCZ, genes obtained improved results with AUC, and AUPRC, of 0.88 and 0.89 respectively.

**FIGURE 6:**
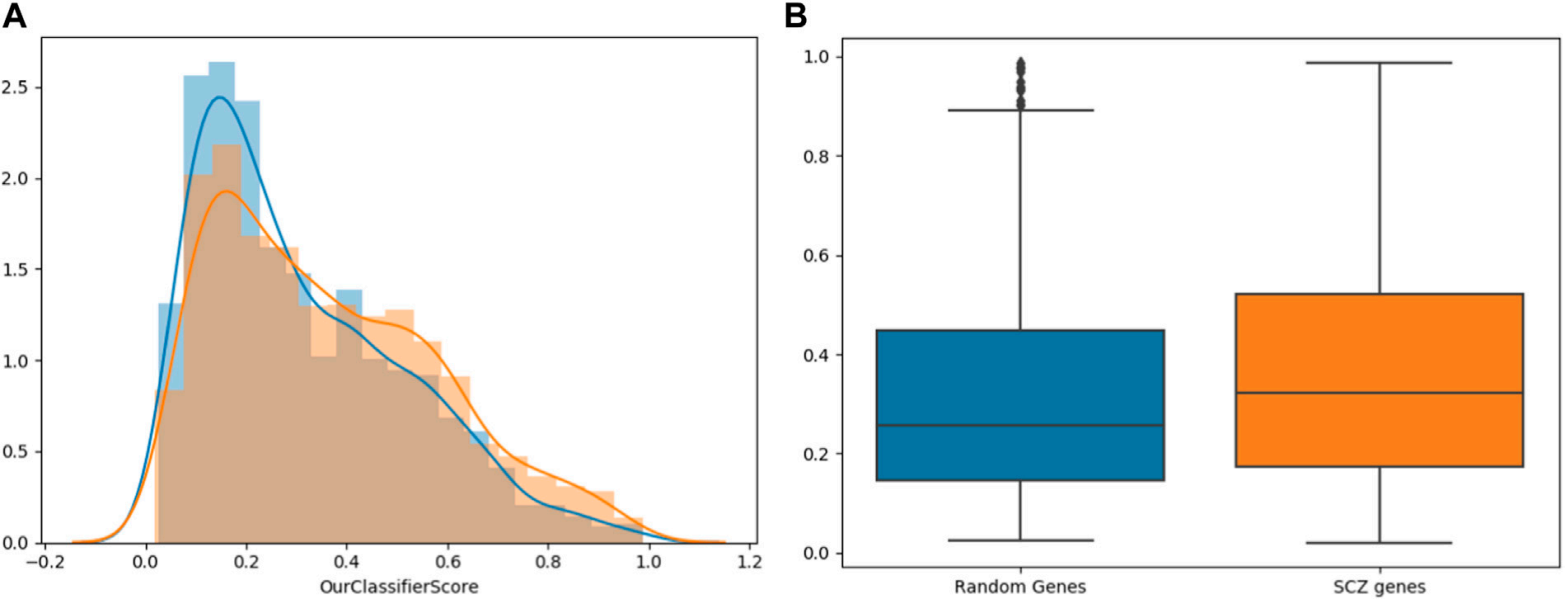
Classifier Predictions on SCZ Genes. The distribution of SCZ gene scores (Orange) versus that of random genes (Blue). The distribution is shown both in histograms **(A)** and in box plots **(B)**.

Next, we checked if our ASD classifier can also predict schizophrenia associated genes. We ran it on genes which are associated with SCZ from a database for Schizophrenia genetic research, SZDB (Wu et al., 2017). Specifically, we focused on 1622 SCZ-associated genes from SZDB with scores higher than 3, and compared their scores to those of the same number of random genes (Figure 6). Wilcoxon signed-rank test showed that the classifier gave the SCZ-associated genes significantly higher scores than the random genes (*p*-value of 2.275e-11).

## Conclusion

We have presented a classification model for disease association. The classifier uses network propagation which enables us to combine and to amplify signals from individual genes, and uses machine learning to allow us to learn from known genes in order to classify new ones. In application to ASD, our classifier attained high accuracy, outperforming the state of the art. We have further shown its applicability to SCZ, which benefits from the similarity between these diseases but also shows the generality of our approach.

Functional enrichment analysis of our proposed candidate ASD genes has pointed to several pathways and processes that have been previously linked to ASD. For instance, neuron cell-cell adhesion, which may contribute to neuroinflammation in ASD (Eve et al., 2022) and participates in neurodevelopmental pathways associated with the disorder (Gandawijaya et al., 2020). Moreover (Bonsi et al., 2022), demonstrated that genes associated with ASD are frequently involved in the structural organization and functional activity of synapses, as evident in our results indicating enriched pathways such as synapse assembly and presynaptic and postsynaptic membrane assembly. This is interesting, as ASD is sometimes regarded as a disorder of connectivity (Mohammad-Rezazadeh et al., 2016), since its genes have both direct and indirect effect on a range of presynaptic and postsynaptic proteins (Bonsi et al., 2022; Yeo et al., 2022).

The fact that our classifier succeeded in predicting Schizophrenia-associated genes may suggest that previously implied association between ASD and SCZ may evolve from connectivity-related issues (Hommer and Swedo, 2015).

## Data Availability

The original contributions presented in the study are included in the article/Supplementary Material, further inquiries can be directed to the corresponding author.

## References

[B1] Abrahams B. S. Arking D. E. Campbell D. B. Mefford H. C. Morrow E. M. Weiss L. A. (2013). SFARI Gene 2.0: a community-driven knowledgebase for the autism spectrum disorders (ASDs). Mol. autism 4 (1), 36–43. 10.1186/2040-2392-4-36 24090431 PMC3851189

[B2] Bonsi P. De Jaco A. Fasano L. Gubellini P. (2022). Postsynaptic autism spectrum disorder genes and synaptic dysfunction. Neurobiol. Dis. 162, 105564. 10.1016/j.nbd.2021.105564 34838666

[B3] Brueggeman L. Koomar T. Michaelson J. J. (2020). Forecasting risk gene discovery in autism with machine learning and genome-scale data. Sci. Rep. 10 (1), 4569. 10.1038/s41598-020-61288-5 32165711 PMC7067874

[B4] Duda M. Zhang H. Li H. D. Wall D. P. Burmeister M. Guan Y. (2018). Brain-specific functional relationship networks inform autism spectrum disorder gene prediction. Transl. psychiatry 8 (1), 56. 10.1038/s41398-018-0098-6 29507298 PMC5838237

[B5] Erten S. Bebek G. Ewing R. M. Koyutürk M. (2011). DADA: degree-aware algorithms for network-based disease gene prioritization. BioData Min. 4, 19. 10.1186/1756-0381-4-19 21699738 PMC3143097

[B6] Eve M. Gandawijaya J. Yang L. Oguro-Ando A. (2022). Neuronal cell adhesion molecules may mediate neuroinflammation in autism spectrum disorder. Front. psychiatry/Front. Res. Found. 13, 842755. 10.3389/fpsyt.2022.842755 PMC9051034 35492721

[B7] Flickr F. (2023). Us on (no date) about Autism. Available at: https://www.nichd.nih.gov/health/topics/autism/conditioninfo (Accessed February 28, 2023).

[B8] Gandal M. J. Zhang P. Hadjimichael E. Walker R. L. Chen C. Liu S. (2018). Transcriptome-wide isoform-level dysregulation in ASD, schizophrenia, and bipolar disorder. Science 362 (6420), eaat8127. Available at:. 10.1126/science.aat8127 30545856 PMC6443102

[B9] Gandawijaya J. Bamford R. A. Burbach J. P. H. Oguro-Ando A. (2020). Cell adhesion molecules involved in neurodevelopmental pathways implicated in 3p-deletion syndrome and autism spectrum disorder. Front. Cell. Neurosci. 14, 611379. 10.3389/fncel.2020.611379 33519384 PMC7838543

[B10] Haddad Derafshi B. Danko T. Chanda S. Batista P. J. Litzenburger U. Lee Q. Y. (2022). The autism risk factor CHD8 is a chromatin activator in human neurons and functionally dependent on the ERK-MAPK pathway effector ELK1. Sci. Rep. 12 (1), 22425. 10.1038/s41598-022-23614-x 36575212 PMC9794786

[B11] Hommer R. E. Swedo S. E. (2015). Schizophrenia and autism-related disorders. Schizophr. Bull. 41 (2), 313–314. 10.1093/schbul/sbu188 25634913 PMC4332956

[B12] Huang G. Osorio D. Guan J. Ji G. Cai J. J. (2020). Overdispersed gene expression in schizophrenia. NPJ Schizophr. 6 (1), 9. 10.1038/s41537-020-0097-5 32245959 PMC7125213

[B13] Krishnan A. Zhang R. Yao V. Theesfeld C. L. Wong A. K. Tadych A. (2016). Genome-wide prediction and functional characterization of the genetic basis of autism spectrum disorder. Nat. Neurosci. 19 (11), 1454–1462. 10.1038/nn.4353 27479844 PMC5803797

[B14] Lin Y. Afshar S. Rajadhyaksha A. M. Potash J. B. Han S. (2020). A machine learning approach to predicting autism risk genes: validation of known genes and discovery of new candidates. Front. Genet. 11, 500064. 10.3389/fgene.2020.500064 33133139 PMC7513695

[B15] Liu L. Lei J. Sanders S. J. Willsey A. J. Kou Y. Cicek A. E. (2014). DAWN: a framework to identify autism genes and subnetworks using gene expression and genetics. Mol. autism 5 (1), 22–18. 10.1186/2040-2392-5-22 24602502 PMC4016412

[B16] Liu X. (2012). Classification accuracy and cut point selection. Statistics Med. 31 (23), 2676–2686. 10.1002/sim.4509 22307964

[B17] Maenner M. J. (2021). ‘Prevalence and characteristics of autism spectrum disorder among children aged 8 Years — autism and developmental Disabilities monitoring network, 11 sites, United States, 2018’, morbidity and mortality weekly report. Surveill. Summ., 70. Available at:. 10.15585/mmwr.ss7011a1 PMC8639024 34855725

[B18] Mohammad-Rezazadeh I. Frohlich J. Loo S. K. Jeste S. S. (2016). Brain connectivity in autism spectrum disorder. Curr. Opin. neurology 29 (2), 137–147. 10.1097/wco.0000000000000301 PMC4843767 26910484

[B19] Parikshak N. N. Swarup V. Belgard T. G. Irimia M. Ramaswami G. Gandal M. J. (2016). Genome-wide changes in lncRNA, splicing, and regional gene expression patterns in autism. Nature 540 (7633), 423–427. 10.1038/nature20612 27919067 PMC7102905

[B20] Raudvere U. Kolberg L. Kuzmin I. Arak T. Adler P. Peterson H. (2019). g:Profiler: a web server for functional enrichment analysis and conversions of gene lists (2019 update). Nucleic acids Res. 47 (W1), W191–W198. 10.1093/nar/gkz369 31066453 PMC6602461

[B21] Sanders S. J. He X. Willsey A. Ercan-Sencicek A. Samocha K. Cicek A. (2015). Insights into autism spectrum disorder genomic architecture and biology from 71 risk loci. Neuron 87 (6), 1215–1233. 10.1016/j.neuron.2015.09.016 26402605 PMC4624267

[B22] Satterstrom F. K. Kosmicki J. A. Wang J. Breen M. S. De Rubeis S. An J. Y. (2020). Large-scale exome sequencing study implicates both developmental and functional changes in the neurobiology of autism. Cell. 180 (3), 568–584.e23. 10.1016/j.cell.2019.12.036 31981491 PMC7250485

[B23] Signorini L. F. Almozlino T. Sharan R. (2021). ANAT 3.0: a framework for elucidating functional protein subnetworks using graph-theoretic and machine learning approaches. BMC Bioinforma. 22 (1), 526–6. 10.1186/s12859-021-04449-1 PMC8555137 34706638

[B24] Sun W. Poschmann J. Cruz-Herrera del Rosario R. Parikshak N. N. Hajan H. S. Kumar V. (2016). Histone acetylome-wide association study of autism spectrum disorder. Cell. 167 (5), 1385–1397.e11. 10.1016/j.cell.2016.10.031 27863250

[B25] Walsh W. J. (2002). Metallothionein and autism’, and autism 2nd edn. Naperville, IL: pfeiffer.

[B26] Walsh W. J. Usman A. Tarpey J. (2001). Disordered metal metabolism in a large autism population. walshinstitute.Org. Available at: http://www.walshinstitute.org/uploads/1/7/9/9/17997321/disordered_metal_metabolism_in_a_large_autism_population.pdf (Accessed August 28, 2023).

[B27] Wang X. Zhou B. (2010). Dietary zinc absorption: a play of Zips and ZnTs in the gut. IUBMB life 62 (3), 176–182. 10.1002/iub.291 20120011

[B28] Wong C. C. Y. Smith R. G. Hannon E. Ramaswami G. Parikshak N. N. Assary E. (2019). Genome-wide DNA methylation profiling identifies convergent molecular signatures associated with idiopathic and syndromic autism in post-mortem human brain tissue. Hum. Mol. Genet. 28 (13), 2201–2211. 10.1093/hmg/ddz052 31220268 PMC6602383

[B29] Wu Y. Yao Y.-G. Luo X.-J. (2017). SZDB: a database for schizophrenia genetic research. Schizophr. Bull. 43 (2), 459–471. 10.1093/schbul/sbw102 27451428 PMC5605257

[B30] Yasuda H. Yoshida K. Yasuda Y. Tsutsui T. (2011). Infantile zinc deficiency: association with autism spectrum disorders. Sci. Rep. 1, 129. 10.1038/srep00129 22355646 PMC3216610

[B31] Yeo X. Y. Lim Y. T. Chae W. R. Park C. Park H. Jung S. (2022). Alterations of presynaptic proteins in autism spectrum disorder. Front. Mol. Neurosci. 15, 1062878. 10.3389/fnmol.2022.1062878 36466804 PMC9715400

[B32] Zhang C. Shen Y. (2017). A cell type-specific expression signature predicts haploinsufficient autism-susceptibility genes. Hum. Mutat. 38 (2), 204–215. 10.1002/humu.23147 27860035 PMC5865588

